# Risk factors for diabetic foot ulcers in metreleptin naïve patients with lipodystrophy

**DOI:** 10.1186/s40842-021-00132-9

**Published:** 2021-10-01

**Authors:** O Saydam, B Ozgen Saydam, SC Adiyaman, M Sonmez Ince, MA Eren, FE Keskin, H Bilen, M Dagdeviren, S Kaya, G Akinci, A Balci, C Altay, F Bayraktar, EA Oral, B Akinci

**Affiliations:** 1 Division of Cardiovascular Surgery, Izmir Tepecik Training and Research Hospital, Izmir, Turkey; 2 Division of Endocrinology and Metabolism, Dokuz Eylul University Faculty of Medicine, Inciralti, Izmir, Turkey; 3 Department of Internal Medicine, William Beaumont Royal Oak Hospital, MI Royal Oak, USA; 4 Division of Endocrinology and Metabolism, Harran University Faculty of Medicine, Sanliurfa, Turkey; 5 Division of Endocrinology and Metabolism, Demiroglu Bilim University Faculty of Medicine, Istanbul, Turkey; 6 Division of Endocrinology and Metabolism, Ataturk University Training and Research Hospital, Erzurum, Turkey; 7 Division of Endocrinology and Metabolism, Kecioren Training and Research Hospital, Ankara, Turkey; 8 Department of Internal Medicine, Gulhane Training and Research Hospital, Ankara, Turkey; 9 Division of Pediatric Neurology, Behcet Uz Children’s Hospital, Izmir, Turkey; 10 Department of Neurology, University of Michigan, Ann Arbor, MI USA; 11 Department of Radiology, Dokuz Eylul University Faculty of Medicine, Izmir, Turkey; 12 Brehm Center for Diabetes Research and Division of Metabolism, Endocrinology and Diabetes, University of Michigan, 1000 Wall Street, 48105 Ann Arbor, MI USA

**Keywords:** Diabetes, Foot ulcer, Lipodystrophy, Neuropathy, Peripheral artery disease

## Abstract

**Aim:**

Patients with lipodystrophy are at high risk for chronic complications of diabetes. Recently, we have reported 18 diabetic foot ulcer episodes in 9 subjects with lipodystrophy. This current study aims to determine risk factors associated with foot ulcer development in this rare disease population.

**Methods:**

Ninety metreleptin naïve patients with diabetes registered in our national lipodystrophy database were included in this observational retrospective cohort study (9 with and 81 without foot ulcers).

**Results:**

Patients with lipodystrophy developing foot ulcers had longer diabetes duration (*p* = 0.007), longer time since lipodystrophy diagnosis (*p* = 0.008), and higher HbA1c levels (*p* = 0.041). Insulin use was more prevalent (*p* = 0.003). The time from diagnosis of diabetes to first foot ulcer was shorter for patients with generalized lipodystrophy compared to partial lipodystrophy (*p* = 0.036). Retinopathy (*p* < 0.001), neuropathy (*p* < 0.001), peripheral artery disease (*p* = 0.001), and kidney failure (*p* = 0.003) were more commonly detected in patients with foot ulcers. Patients with foot ulcers tended to have lower leptin levels (*p* = 0.052). Multiple logistic regression estimated significant associations between foot ulcers and generalized lipodystrophy (OR: 40.81, 95% CI: 3.31–503.93, *p* = 0.004), long-term diabetes (≥ 15 years; OR: 27.07, 95% CI: 2.97–246.39, *p* = 0.003), and decreased eGFR (OR: 13.35, 95% CI: 1.96–90.67, *p* = 0.008).

**Conclusions:**

Our study identified several clinical factors associated with foot ulceration among patients with lipodystrophy and diabetes. Preventive measures and effective treatment of metabolic consequences of lipodystrophy are essential to prevent the occurrence of foot ulcers in these high-risk individuals.

## Introduction

Lipodystrophy is a rare disease characterized by generalized or partial loss of subcutaneous and visceral fat [1]. The management of metabolic abnormalities in lipodystrophy can be quite compelling. These metabolic abnormalities are usually severe and may result in end-organ complications (e.g. nephropathy, retinopathy, neuropathy, acute pancreatitis, hepatic cirrhosis, cardiovascular disease) at relatively younger ages. Severe insulin resistance due to lipodystrophy leads to “difficult to treat” diabetes associated with early onset of diabetic complications [2]. Metreleptin, a European Medicines Agency (EMA) and Food and Drug Administration (FDA) approved treatment, offers significant metabolic benefits to patients with lipodystrophy [3, 4]. However, metreleptin was not available until very recently in Turkey. Still, only selected patients with generalized lipodystrophy (GL) could qualify for reimbursement under certain circumstances.

We have recently reported 18 diabetic foot ulcer (DFU) episodes that occurred in 9 Turkish patients with various forms of lipodystrophy [5]. DFUs are a serious complication of diabetes, a major cause of lower-limb amputations [6]. Patients with DFUs have a greater risk of death than those with diabetes but no DFUs [7]. Diabetic neuropathy, a relatively common complication of diabetes, is often a predisposing factor to ulceration and amputation [8]. Peripheral arterial disease (PAD) is another leading etiology involved in the pathogenesis of DFUs [9].

In this observational retrospective cohort study, we aimed to determine clinical risk factors for DFUs in metreleptin naïve patients with lipodystrophy and diabetes.

## Materials and methods

The study includes data from 90 patients with lipodystrophy and diabetes who were admitted to one of our centers between January 2008 and January 2020. All patients were metreleptin naïve at the time of final visit data collection. The study was approved by the Dokuz Eylul University ethical review board.

Data were retrieved from 206 patients with lipodystrophy registered in the national lipodystrophy database. To determine risk factors associated with DFU development in lipodystrophy, clinical characteristics of the 9 lipodystrophy patients developing DFUs were compared to the rest of the national lipodystrophy cohort. Patients were excluded if they were not diabetic yet or no recent follow-up was available. Diabetes was defined according to the recommendations of the American Diabetes Association [10]. Also, patients with human immunodeficiency virus (HIV)-associated lipodystrophy, local lipodystrophy, progeria-associated lipodystrophy, and complex syndromes were not included. Visits were excluded if they happened after the initiation of metreleptin. Otherwise, all lipodystrophy patients with diabetes were included in an unbiased manner. Finally, data from 81 patients were available for comparison.

A standard form was used to collect information regarding patients’ characteristics, medical history, and physical examination findings. Patients were screened for end-organ complications. Serum biochemistry, HbA1c, urinary protein, and leptin levels were determined according to standard methods with appropriate quality control and quality assurance procedures. In patients with DFU, hospital records were reviewed to confirm the baseline wound characteristics and collect data regarding the duration of antibiotic treatment, hospital stay, and the outcome of the episode. The site of the ulcer was noted. Depth of the ulcer was determined by inspection, with the additional use of a sterile probe if indicated. Plain x-ray films were taken. Magnetic resonance imaging (MRI) of the extremity was performed if needed.

The presence of diabetic neuropathy was evaluated by questioning symptoms of neuropathy, neurological examination, and monofilament test (10 g). Lower extremity arterial circulation was determined clinically by palpation of the peripheral pulses and conventional Doppler examination if needed. For patients with hemodynamically significant findings in Doppler examination, computed tomography (CT) angiography, magnetic resonance angiography or conventional angiography were performed as indicated.

Patients with DFUs received a standard treatment that included wound care, bed rest, proper offloading, parenteral antibiotics, and debridement or amputation (minor or major) when indicated. Extensive callus and hyperkeratotic skin surrounding the wound edge and necrotic tissues were removed by debridement. Footwear was tailored for each patient. Antibiotics were given if an infection was detected based on the Infectious Diseases Society of America guidelines [11]. Antibiotic treatment was guided by culture results, which were obtained by deep-needle aspiration, bone biopsy, or curettage of the ulcer.

Statistical analyses were performed using SPSS version 22.0 (SPSS Inc., Chicago, IL, USA). The normality was assessed by the Kolmogorov–Smirnov test. Because of the small number of subjects with lipodystrophy developing DFU and the skewed distribution of the data, non-parametric tests were preferred. Categorical parameters were compared using Fisher’s exact test. Mann Whitney U test was used to compare continuous variables. Receiver-operating characteristic (ROC) analysis was used to determine potential predictors of the development of DFUs among patients with lipodystrophy. The optimal cut-points were estimated using the Youden indices. Odds ratios with 95 % confidence interval (CI) were calculated to investigate factors associated with DFU among patients with lipodystrophy using a logistic regression model. Models were adjusted for age, gender, and smoking history. Data were then treated in a time to event analysis approach; the reference date was the year of diagnosis for diabetes. Kaplan Meier curves were plotted for DFUs to compare between generalized and partial lipodystrophy groups. Data were presented as median and 25th -75th percentiles. Two-tailed *p*-value < 0.05 was considered statistically significant.

## Results

### Description of DFUs in patients with lipodystrophy

Among 90 metreleptin naïve patients with lipodystrophy and diabetes, DFUs were observed in 9 subjects. These foot ulcers have been briefly reported recently [5]. A more detailed description of these ulcer episodes in individual patients are presented below.

**Fig. 1:**
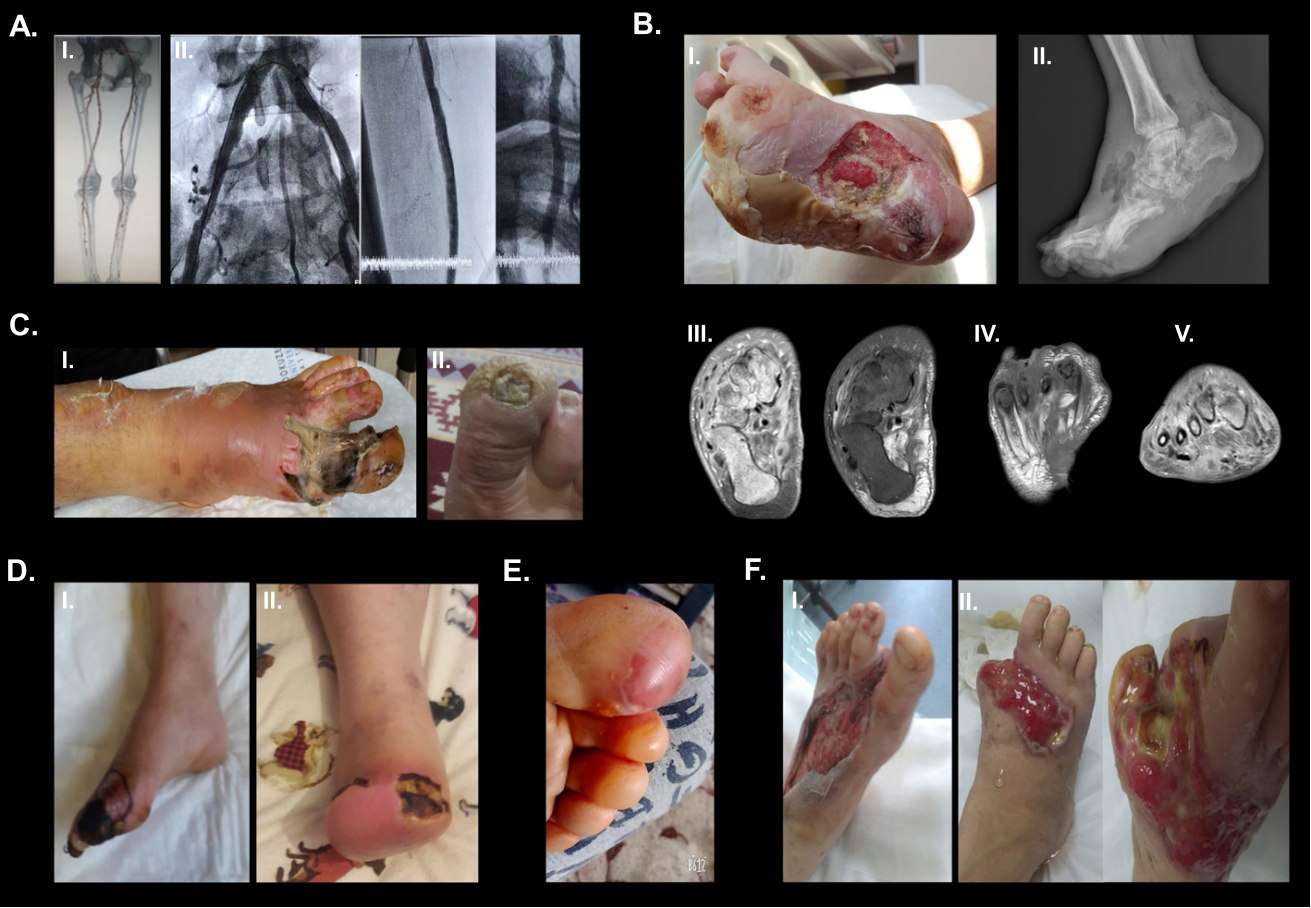
** A.** CT angiography and coventional angiography images of patient-1 [CGL1; *AGPAT2* homozygous p.R68X (c.202 C > T)]. 3D computed tomography reconstruction of lower abdomen and extremity arteries showing severe stenosis in the right common iliac artery, superficial femoral artery (SFA), and P1 segment of the popliteal artery (PA) (I). High pressure 5-mm drug-eluting balloon (DEB) angioplasty was performed to the P1 segment of the PA. High pressure plain old balloon angioplasty (POBA) followed with 6-mm self-expanding nitinol stent implantation and post dilatation with 6mm POBA was performed to the SFA. A 10-mm balloon-expandable bare metal stent was deployed to the right common iliac artery beginning from the orifice (II). The ulcer healed weeks after the revascularization procedure. **B.** Patient-2 [CGL1; *AGPAT2* homozygous IVS5-2 A > C (c.662-2 A > C)] had several episodes of diabetic foot ulcer (DFU). Here, her 6th episode is shown. Figure 1B (I) shows a deep infected predominantly neuropathic ulcer on the medial aspect of the right foot with a total infected area of 200 cm^2^, disseminated osteomyelitis, and soft tissue abscess. Plain lateral foot radiogram (II) shows rocker-bottom foot deformity, gas in soft tissues associated with gas gangrene, disseminated osteolysis, bone deformation and destruction. T1-weighted axial MR images (III) show distal metatarsal bone marrow edema, joint inflammation and soft tissue edema. Postcontrast T1-weighted fat-saturated axial (IV) and coronal (V) MR images display diffuse contrast enhancement of bone marrow, and soft tissue edema which is compatible with ostomyelitis and cellulitis. A soft tissue abscess formation is shown. Wound culture identified several pathogens over time including Enterococcus spp., ESBL-producing Escherichia coli, and Candida albicans. The DFU did not heal despite broad-spectrum antibiotics including ampicillin-sulbactam plus ciprofloxacin (81 days), meropenem (17 days) and teicoplanin (31 days), aggressive debridement, predilatation with the plain old balloon angioplasty followed by DEB angioplasty to the right SFA, and vacuum assisted closure (VAC) therapy was performed. She underwent a below the knee amputation; however, she is still being treated for wound infection at the amputation site. **C.** A grade 4 ulcer (8 × 4 cm) on the left big toe and first metatarsus, spreading to the neighboring forefoot areas which is complicated with osteomyelitis and necrosis [I; Patient-3: CGL1; *AGPAT2* homozygous p.E229X (c.685G > T)]. Wound culture identified Streptococcus spp., and she received clindamycin and ciprofloxacin P.O. for 18 days. The vascular assessment identified left popliteal artery stenosis and above-the-knee amputation was performed. She also received VAC and hyperbaric oxygen post-amputation. She later developed a grade 1 neuropathic ulcer on the right big toe (II). She had accompanying onychomycosis. In the same period, she developed multiple bullae in her both hands located on the right third and left second and third fingers. These lesions healed with local wound care and antifungal treatment. **D.** A necrotic forefoot ulcer covering the entire right big toe, index toe and middle toe (I; Patient-7: APL). The ulcer is complicated with osteomyelitis and necrosis. Infected tissue necrosis to the stump extending the suture line (II). **E.** Bullous lesions on the plantar surfaces of toes (big, index, middle and fourth toes in the left foot; varying between 1 × 1 and 3 × 3 cm in size. [Patient-8: CGL1; *AGPAT2* homozygous p.D180PfsX5 (c.538_539delGA)]. **F.** A large neuropathic ulcer (20 × 5 cm) predominantly affecting the dorsal aspect of the forefoot and midfoot (I) in patient-9 [FPLD2; *LMNA* heterozygous p.R482W (c.144 C > T)]. She received parenteral meropenem for 21 days followed by amoxicillin-clavulanic acid and ciprofloxacin P.O. for 6 months, and the ulcer healed. Later, she presented with another neuropathic ulcer that developed in her right big toe but widespread into her forefoot and even midfoot. The ulcer was further complicated with cellulitis, soft tissue abscess, osteomyelitis, and local gangrene. She was treated with parenteral piperacillin and tazobactam and linezolid (16 days) which was followed by linezolid monotherapy (P.O. for 14 days). She underwent a toe amputation but the ulcer has not healed yet despite wound care (II)

Patient-1 developed a small (1 × 0.5 cm) grade 1 ulcer on the dorsum of his right foot. The patient had diabetic neuropathy but the absence of typical features of a neuropathic ulcer such as an ulcer forming on the pressure points on the limb or callus formation prompted us to search for PAD. CT angiography revealed heavy calcifications in both extremity arteries and severe stenosis in the right common iliac artery (CIA), right superficial femoral artery (SFA), and P1 segment of the right popliteal artery (PA) (Fig. 1 A).

Patient-2 had multiple episodes of predominantly neuropathic type ulcers. She was first presented with a 3 × 2 cm ulcer located on the right middle toe complicated with osteomyelitis and local gangrene. The vascular assessment revealed non-significant stenosis in the right CIA and bilateral chronic occlusions in the posterior tibial artery (PTA). The revascularization of the right PTA was unsuccessful due to severe calcifications. The ulcer did not heal despite off-loading and treatment with ampicillin/sulbactam (52 days) followed by piperacillin/tazobactam (9 days), tigecycline/ciprofloxacin (17 days) and moxifloxacin (14 days). This episode required a minor amputation. In the subsequent year, she developed an ulcer on the dorsum of the big toe (1 × 1 cm) complicated with cellulitis spreading to the right anterior tibia. Cellulitis healed with ampicillin/sulbactam (18 days) followed by clindamycin/ciprofloxacin (4 days), and the ulcer improved with local wound care. However, she was admitted a few months later with a purulent section coming from the ulcer and a newly developed ulcer under the little toe. Her ulcer on the right toe was bigger this time (3 × 3 cm). Osteomyelitis was detected. Culture work-up revealed multiple organisms. The ulcer healed after vacuum-assisted closure (VAC) therapy along with local debridement and prolonged antibiotic treatment with clindamycin/ciprofloxacin (85 days), followed by imipenem/linezolid (15 days) and ertapenem/daptomycin (30 days). Several months later, she presented with multiple small neuropathic ulcers (ranging from 1 to 3 cm) located under the right metatarsal head complicated with osteomyelitis. These ulcers healed after debridement and curettage of the first metatarsal bone along with antibiotics (clindamycin/ciprofloxacin for 57 days). Her fifth episode occurred on her left foot under the big toe. This neuropathic ulcer required prolonged antibiotic treatment for osteomyelitis including ampicillin/sulbactam (206 days) followed by clindamycin/ciprofloxacin 116 days). A few years later, she presented with a severe DFU in the right foot (Fig. 1B). CT angiography revealed heavy calcifications and severe stenosis in the right SFA. As predilatation with the plain old balloon angioplasty (POBA) under nominal pressure (10 atm) was insufficient, high-pressure drug-eluting balloon (DEB) angioplasty was performed under 20 atm for 3 min to the right SFA. After the successful vascular intervention, the patient underwent below-the-knee amputation.

Patient-3 presented with a gangrenous ulcer on her left big toe and first metatarsus, spreading to the forefoot, which was complicated with cellulitis of surrounding tissues and osteomyelitis (Fig. 1 C-I). Left popliteal artery stenosis was detected and the patient underwent a below-the-knee amputation. About 2 years later, she developed a bullous lesion on the lateral malleolus of the right leg (2 × 2 cm) which was complicated with mild superficial cellulitis. Non-significant right proximal superficial artery stenosis was detected. The lesion healed with local wound care. A few months after, she presented with a grade 1 neuropathic ulcer surrounded by callous in her right big toe (Fig. 1 C-II).

Patient-4 developed a 5 × 3 cm ulcer on the plantar aspect of the first right metatarsal head which was complicated with cellulitis and abscess formation. Posterior and anterior tibial artery occlusions were detected. The ulcer healed after treatment with debridement and parenteral clindamycin and ciprofloxacin for 14 days.

Patient-5 presented with a neuropathic ulcer on the plantar surface of the right big toe complicated with cellulitis, abscess formation, osteomyelitis, and local necrosis. The infection progressed despite treatment with systemic antibiotics and the episode resulted in the amputation of the right toe.

Patient-6 developed a predominantly neuropathic ulcer on the plantar aspect of the left big and fourth toes which was complicated with abscess formation, osteomyelitis, and necrosis. Wound culture showed multiple microorganisms over time. She was treated with parenteral ciprofloxacin/piperacillin/tazobactam (7 days) followed by amoxicillin/clavulanic acid and ciprofloxacin (15 days). The episode resulted in auto-amputation of the two toes but the infection did not heal. The patient was discharged with wound care but readmitted several months later with disseminated infection and died due to sepsis despite treatment with broad-spectrum antibiotics.

Patient-7 presented with a necrotic forefoot ulcer complicated with osteomyelitis (Fig. 1D-I). The patient was treated with transmetatarsal amputation first, but she developed infected tissue necrosis after her first surgery (Fig. 1D-II) and underwent below-knee amputation.

Patient-8 was admitted because of bullous lesions on the plantar surfaces of toes (Fig. 1E). She received 7 days of amoxicillin-clavulanic acid, and the lesions healed after minor debridement.

Patient-9 first presented with a large neuropathic ulcer on the dorsal aspect of the forefoot and midfoot, which was complicated with osteomyelitis (Fig. 1 F-I). This patient later presented with another neuropathic ulcer and underwent a toe amputation (Fig. 1 F-II).

### Comparison of patients with lipodystrophy developing DFU to those without DFU

Clinical characteristics of lipodystrophy patients developing DFUs are presented in Table 1. The characteristics of patients with lipodystrophy developing DFUs were compared to lipodystrophy patients with diabetes but no DFUs (Table 2). Patients with DFUs had longer disease durations for lipodystrophy and diabetes. Their glycemic control was worse. All patients developing DFUs were on insulin, while half of lipodystrophy patients without DFUs were still being treated with oral antidiabetics, another indicator of the presence of more severe diabetes in patients developing foot ulcers. Retinopathy, neuropathy and PAD were more commonly detected among patients with lipodystrophy developing DFUs. Patients with DFUs tended to have lower leptin levels. Also, patients with DFUs were more likely to have decreased estimated glomerular filtration rate (eGFR).

**Table 1:** Characteristics of patients with lipodystrophy developing diabetic foot ulcers

	Patient 1	Patient 2	Patient 3	Patient 4	Patient 5	Patient 6	Patient 7	Patient 8	Patient 9
Current age	Died at age 66	35	26	55	25	Died at age 26	66	33	38
Gender (F/M)	M	F	F	F	F	F	F	F	F
Type of lipodystrophy	CGL1	CGL1	CGL1	FPLD1	CGL1	CGL2	APL	CGL1	FPLD2
Pathogenic variant	*AGPAT2* Homozygous p.R68X (c.202 C > T)	*AGPAT2* Homozygous IVS5-2 A > C (c.662-2 A > C)	*AGPAT2* Homozygous p.E229X (c.685G > T)	Negative	*AGPAT2* Homozygous p.K216X (c.646 A > T)	*BSCL2* Homozygous p.Q94X (c.280 C > T)	No genetic testing done	*AGPAT2* Homozygous p.D180PfsX5 (c.538_539delGA)	*LMNA* Heterozygous p.R482W (c.144 C > T)
Age at first episode	66	27	24	50	24	24	65	32	34
Complications of lipodystrophy and diabetes	Nephropathy Neuropathy CAD PAD	Retinopathy Nephropathy Neuropathy CAD PAD	Nephropathy Neuropathy PAD	Retinopathy Neuropathy PAD	Retinopathy Nephropathy Neuropathy	Retinopathy Nephropathy Neuropathy PAD	Retinopathy Nephropathy Neuropathy PAD	Nephropathy Neuropathy	Retinopathy Neuropathy
Leptin (ng/mL)	0.76	0.85	0.38	12.57	0.79	0.10	N/A	< 0.10	0.94
Adiponectin (ng/mL)	1.80	0.54	1.50	4.29	0.09	27.21	N/A	N/A	5.12
Treatment of lipodystrophy related metabolic diseases and other medications	Basal-bolus insulin Atorvastatin Amlodipine Furosemide α-lipoic acid Gabapentin Clopidogrel ASA	Basal-bolus insulin Metformin Atorvastatin Nebivolol Zofenopril Tacrolimus Mycophenolate mofetil ASA Cilostazol Clopidogrel	Basal-bolus insulin Metformin Pioglitazone Fenofibrate Cilostazol Clopidogrel	Basal-bolus insulin Metformin Pregabalin	Basal-bolus insulin Metformin Fenofibrate	Basal-bolus insulin Calcitriol Furosemide Propranolol Cilostazol ASA	Premix insulin Sitagliptin Atorvastatin	Basal-bolus insulin Metformin Pioglitazone Fenofibrate	Basal-bolus insulin Metformin Fenofibrate Citalopram Adalimumab Metoprolol

*AGPAT* 1-acylglycerol-3-phosphate O-acyltransferase, *APL* acquired partial lipodystrophy, *ASA* acetylsalicylic acid, *BSCL2* Berardinelli-Seip congenital lipodystrophy type 2, *CAD* coronary artery disease, *CGL* congenital generalized lipodystrophy, *F* female, *FPLD* familial partial lipodystrophy, *LMNA* lamin A/C, *M* male, *PAD* peripheral artery disease

**Table 2:** Comparison of characteristics of diabetic lipodystrophy patients with and without foot ulcers

	Patients developing foot ulcer (*n* = 9)	Patients without foot ulcer (*n* = 81)	*p*-value
Current age (years)	34 (26–52)	35 (25–51)	0.652
Gender (F/M)	8/1	64/17	0.681
Smoking (n, %)	3 (33/3 %)	9 (11.1 %)	0.097
Generalized lipodystrophy (n, %)	6 (67 %)	24 (29.6 %)	0.055
The age when lipodystrophy was diagnosed (years)	15 (6–47)	31 (18–47)	0.187
The age when diabetes developed (years)	24 (10–35)	28 (18–38)	0.253
Time from the diagnosis of lipodystrophy^a^ (years)	19 (2–20)	2 (1–5)	0.008
Duration of diabetes (years)^a^	17 (15–22)	9 (4–14)	0.007
Hypertension (n, %)	3 (33 %)	34 (42 %)	0.732
Retinopathy (n, %)	6 (67 %)	8 (10 %)	< 0.001
Neuropathy (n, %)	9 (100 %)	20 (25 %)	< 0.001
Insulin use (n, %)	9 (100 %)	39 (49 %)	0.003
Peripheral artery disease (n, %)	4 (44 %)	2 (3 %)	0.001
Cardiac disease (n, %)	3 (33 %)	21 (26 %)	0.696
eGFR < 60 ml/min/1.73 m^2^ (n, %)	3 (33 %)	5 (6 %)	0.031
eGFR < 15 ml/min/1.73 m^2^ (n, %)	3 (33 %)	1 (1 %)	0.003
Glucose (mg/dL)	184 (163–203)	155 (115–201)	0.116
HbA1c (%)	9.1 (7.8–11.6)	8.0 (6.4–9.5)	0.041
Triglyceride (mg/dL)	690 (342–1048)	416 (243–931)	0.161
Total cholesterol (mg/dL)	213 (176–235)	207 (175–238)	0.900
LDL cholesterol (mg/dL)	105 (66–111)	107 (84–131)	0.585
HDL cholesterol (mg/dL)	28 (25–36)	32 (25–38)	0.496
Leptin (ng/mL)	0.78 (0.24–0.90)	3.01 (0.70–6.23)	0.052

^a^Defined as the time from the diagnosis to the date of the last follow-up. Patients without diabetes are excluded. *eGFR* estimated glomerular filtration rate, *F* female, *HDL-C* high-density lipoprotein cholesterol, *LDL-C* low-density lipoprotein cholesterol, *M* male

**Fig. 2:**
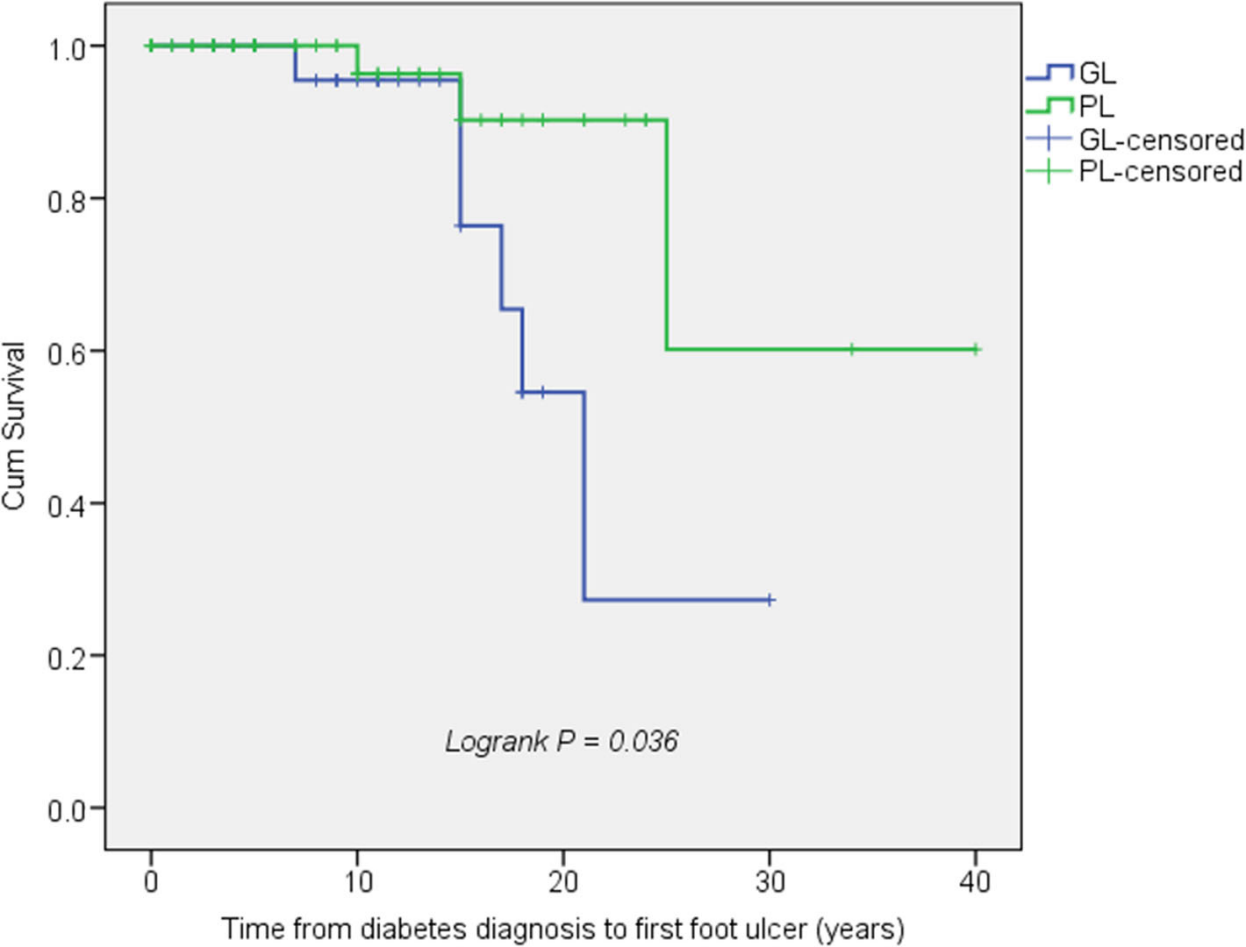
The development of diabetic foot ulcers by type of lipodystrophy. Kaplan-Meier survival curves show a comparison of patients with generalized and partial lipodystrophy. The X-axis shows the duration of time from diagnosis of diabetes to the onset of first diabetic foot ulcer. The outcome is censored on the date of the last follow-up if there is no diabetic foot ulcer episode

The time from diagnosis of diabetes to first DFU was significantly shorter for patients with GL compared to partial lipodystrophy (Fig. 2). ROC analysis revealed the time from the clinical diagnosis of lipodystrophy, duration of diabetes and HbA1c as predictors of DFUs (data controlled for age, gender, and smoking history). Although leptin level had an area under the curve (AUC) value greater than 0.7, its predictive value did not reach statistical significance (Table 3).

**Table 3:** Predictors of the development of diabetic foot ulcers among patients with lipodystrophy, ROC analysis

	AUC	95% CI
Age (years)	0.546	0.347–0.744
Time from the clinical diagnosis of lipodystrophy^a^ (years)	0.767	0.581–0.953^b^
Duration of diabetes^a^ (years)	0.774	0.639–0.910 ^b^
Triglycerides (mg/dL)	0.643	0.479–0.807
HbA1c (%)	0.709	0.543–0.875^b^
Leptin (ng/mL)	0.711	0.516–0.906

^a^Disease duration is defined as the time from the clinical diagnosis of lipodystrophy to the date of last follow-up. Diabetes duration is defined as the time from the diagnosis of diabetes to the date of last follow-up. ROC analysis is used to determine potential predictors of diabetic foot ulcers among patients with lipodystrophy. *AUC* area under the curve, *CI* confidence interval, *ROC* receiver-operating characteristic. ^b^*p* < 0.05

The multiple logistic regression model estimated significant associations between DFUs and GL (odds ratio (OR): 40.81, 95 %CI: 3.31–503.93, *p* = 0.004), long-term diabetes (≥ 15 years; OR: 27.07, 95 %CI: 2.97–246.39, *p* = 0.003), the amount of time since the clinical diagnosis of lipodystrophy (≥ 11 years; OR: 22.90, 95 %CI: 3.67–143.09, *p* = 0.001), and decreased eGFR (OR: 13.35, 95 %CI: 1.96–90.67, *p* = 0.008), when data were adjusted for age, gender, smoking (Table 4).

**Table 4:** Summary estimates of the development of diabetic foot ulcers among patients with lipodystrophy

	Odds Ratio	95% CI	*p* value
GL	40.81	3.31–503.93	0.004
Diabetes diagnosed 15 years ago or earlier	27.07	2.97–246.39	0.003
Lipodystrophy diagnosed 11 years ago or earlier	22.90	3.67–143.09	0.001
eGFR < 60 ml/min/1.73 m^2^	13.35	1.96–90.67	0.008

^a^Data controlled for age, gender, and smoking. The optimal cut-points for lipodystrophy and diabetes duration were estimated using the Youden method. *GL* generalized lipodystrophy, *eGFR* estimated glomerular filtration rate

## Discussion

Our study revealed several clinical factors associated with foot ulcer development in a lipodystrophy population with diabetes. These factors include long-standing diabetes, poor glycemic control, presence of GL, the time from the clinical diagnosis of lipodystrophy, insulin use, neuropathy, PAD, retinopathy, and diminished renal functions. Although not significant, leptin levels tended to be lower in patients developing DFUs.

Metabolic abnormalities usually progress and result in end-organ complications in lipodystrophy. Diabetes generally develops at relatively early ages and is severe, and most patients need high doses of insulin unless treated with metreleptin [3]. Previous studies have demonstrated high morbidity and mortality rates in lipodystrophy caused by end-organ complications such as acute pancreatitis, liver disease, kidney disease, diabetes-related complications, cardiovascular events, and serious infections leading to sepsis [12–18].

DFUs are a devastating complication of diabetes with significant morbidity and alarmingly high 5-year mortality rates [19]. Our results revealed the development of DFUs at relatively young ages in lipodystrophy due to the early development of diabetes, poor glycemic control, and other potential factors. Neuropathy was involved in all DFUs in our cohort. Distal symmetric polyneuropathy is one of the most important predictors of DFUs [20]. Previously, we showed that patients with lipodystrophy suffered from a variety of neuromuscular disorders, with 41.9 % of them diagnosed with distal symmetrical sensorimotor polyneuropathy (67.4 % in those with diabetes) [21]. Also, PAD was detected in several patients, another important etiologic factor for DFUs [22, 23].

Lipodystrophy, per se, is a risk factor for foot ulcerations; however, our current study identified a high-risk group for foot ulcers among patients with lipodystrophy and diabetes. Besides neuropathy and/or PAD, patients are at greater risk if they have longstanding diabetes, poor glucose control, or kidney complications. Also, retinopathy was more commonly detected in patients developing DFUs. Patients with DFUs were more likely on treatment with insulin. Longstanding diabetes and poor glycemic control are established risk factors for foot lesions in all forms of diabetes [20]. Retinopathy is associated with visual impairment that can lead to an additional risk for repeated traumas. Patients with kidney failure are also known to have an increased risk of ulceration [24]. Among different lipodystrophy types, the risk seems to be the highest for GL. Also, leptin levels tend to be lower in patients developing DFUs. The association between generalized fat loss and increased risk for DFUs can be partly explained by the severity of metabolic abnormalities in patients with GL. However, the extent of subcutaneous fat loss would be an additional potential mechanism contributing to the increased risk of ulceration [25].

Our results highlight the importance of taking preventive measures for patients with lipodystrophy, especially for those who have additional risk factor as shown in our study. These measures include, but are not limited to, patient education, the proper care of the foot such as nail and skin care, the selection of appropriate footwear, smoking cessation, foot self-exam, an annual foot examination to identify high-risk foot conditions, and timely referral to a foot care specialist [26]. Patients with neuropathy may need additional interventions such as footwear to reduce and redistribute plantar pressure, extra-wide shoes or depth shoes or custom-molded shoes for patients with bone deformities, specific education how to detect early problems progressing to DFU, and callus debridement [26, 27].

Achieving good metabolic control can potentially prevent developing foot ulcers and amputations in patients with lipodystrophy. Our study cohort represents a group of metreleptin naïve patients with lipodystrophy in whom achieving metabolic control is quite difficult. Current evidence suggests that metreleptin is efficacious to treat metabolic consequences of lipodystrophy [4, 28, 29] and has the potential to reduce end-organ complications [3] but further studies are needed to confirm whether these metabolic benefits can be translated into improved long-term outcomes such as clinical improvements in end-organ complications or prevention of these complications.

Endovascular treatments can be used to treat PAD in lipodystrophy [30]. Two patients underwent successful endovascular treatments in our cohort. Both patients had severe calcifications, and nominal pressure balloons were not sufficient to treat the stenosis, thus, high-pressure balloon either alone or with self-expandable nitinol stent implantation was used. As persistent calcific vascular lesions may occur in patients with lipodystrophy, devices such as high-pressure balloons, chocolate balloons, and stents should be kept ready on the shelf before the procedures. Also, extra caution should be taken in administering contrast agents and renal function should be monitored as concomitant kidney problems are common in this patient population [15].

There were several limitations of our study. First of all, the sample size was small due to rarity of lipodystrophy syndromes. Second, additional factors may contribute to high risk for DFUs in lipodystrophy that include consequences of specific molecular etiologies and progeria, but patients with progeroid features were excluded in our study. Nevertheless, our study has a too small sample size to elucidate whether specific genetic variants are associated with risk factors leading to DFUs or may exert a direct effect via mechanisms such as decreased levels of nitric oxide and increased activity of proinflammatory status [31]. Finally, we should note that most asymptomatic patients without DFUs did not undertake routine imaging for PAD according to our follow-up algorithm which might have caused bias.

## Conclusions

Our study reveals that several clinical factors can help identify patients with lipodystrophy who are at higher risk for developing DFUs. In addition to neuropathy and/or PAD, patients with lipodystrophy and diabetes are at greater risk due to longstanding diabetes, poor glycemic control, generalized fat loss, and the presence of other complications. These patients represent a very high-risk target population for whom urgent preventive measures should be undertaken. Multidisciplinary preventive measures and early and aggressive treatment of metabolic consequences of lipodystrophy would potentially prevent the occurrence of etiological factors leading to foot ulcer development.

## Data Availability

The datasets analyzed during this study are included in this published article.

## Abbreviations

AGPAT: 1-acylglycerol-3-phosphate O-acyltransferase
APL: Acquired partial lipodystrophy
ASA: Acetylsalicylic acid
AUC: Area under the curve
BSCL2: Berardinelli-Seip congenital lipodystrophy type 2
CAD: Coronary artery disease
CGL: Congenital generalized lipodystrophy
CI: Confidence interval
CIA: Common iliac artery
CT: Computed tomography
DEB: Drug-eluting balloon
DFU: Diabetic foot ulcer
eGFR: Estimated glomerular filtration rate
EMA: European Medicines Agency
F: Female
FDA: Food and Drug Administration
FPLD: Familial partial lipodystrophy
GL: Generalized lipodystrophy
HDL-C: High-density lipoprotein cholesterol
HIV: Human immunodeficiency virus
LDL-C: Low-density lipoprotein cholesterol
LMNA: Lamin A/C
M: Male
MRI: Magnetic resonance imaging
OR: Odds ratio
PA: Popliteal artery
PAD: Peripheral arterial disease
POBA: Plain old balloon angioplasty
PTA: Posterior tibial artery
ROC: Receiver-operating characteristic
SFA: Superficial femoral artery
VAC: Vacuum-assisted closure
